# 高灵敏度脉冲火焰光度检测器的研制与性能测试

**DOI:** 10.3724/SP.J.1123.2026.03027

**Published:** 2026-09-08

**Authors:** Junbin CHEN, Haoyuan CAI, Zhihan DENG, Yujie JIN, Jianhai SUN

**Affiliations:** 1. 中国科学院空天信息创新研究院，北京 100190; 1. Aerospace Information Research Institute，Chinese Academy of Sciences，Beijing 100190，China; 2. 中国科学院大学电子电气与通信工程学院，北京 100049; 2. School of Electronic，Electrical and Communication Engineering，University of Chinese Academy of Sciences，Beijing 100049，China

**Keywords:** 脉冲式火焰光度检测器, 硫延迟发光, 定量重复性, 硫检出限, 线性范围, pulsed flame photometric detector （PFPD）, delayed sulfur emission, quantitative repeatability, sulfur detection limit, linear range

## Abstract

常规火焰光度检测器（FPD）在复杂烃类基体中易受背景发光和火焰波动影响，限制了痕量硫化物的高灵敏、稳定检测。本工作基于硫延迟发光的时间分辨特性，研制了一种脉冲式火焰光度检测器（PFPD），并围绕结构设计、运行参数优化、硫延迟发光响应及仪器性能测试开展系统研究。检测器由燃烧与点火模块、气路模块、光学采集与检测模块、温度控制模块以及机械支撑与密封模块组成。其中，引火室用于建立稳定的周期性点火，样品气体在石英燃烧室内实现脉冲燃烧，产生的硫延迟发光经滤光与导光组件传输后由光电倍增管（PMT）采集。气路模块采用两路电子压力控制单元（EPC）分别调控引火室与燃烧室供气，实现点火条件与分析燃烧条件的解耦调控。在此基础上，建立了“点火基准建立-燃烧室供气参数优化-检测温度与信号增益匹配”的窗口化优化流程。结果表明，将引火室氢气和空气流量分别固定为8.0 mL/min和19.0 mL/min，脉冲火焰稳定且规律，可作为引火室的点火基准；在该基准下，将燃烧室氢气和空气流量分别调节为5.0 mL/min和3.7 mL/min，此时硫响应的峰面积、灵敏度和信噪比（SNR）同步达到最优；检测器温度约150 ℃时，硫延迟发光响应较强；PMT电压在590~710 V范围内可获得较好的SNR提升。采用10 μmol/mol的H₂S标准气体经动态稀释后评价线性范围，并以1.1 μmol/mol的H₂S标准气体开展定量重复性和硫检出限测试。连续7次进样的定量重复性为0.37%，硫检出限为4.1×10⁻^1^³ g/s，线性范围约为2个数量级。该PFPD具有稳定的脉冲燃烧行为和痕量硫定量能力，可为PFPD结构设计、参数优化、硫延迟发光响应和性能测试提供参考。

## Abstract (en)

The conventional flame photometric detector （FPD） is widely used for sulfur-selective gas chromatography. However， the FPD is sensitive to hydrocarbon backgrounds. Its sulfur response also depends strongly on flame stability. These factors limit stable trace sulfur determination in complex matrices. A pulsed flame photometric detector （PFPD） provides a time-resolved approach to this problem. Sulfur emission appears later than most hydrocarbon background emissions after pulsed combustion. A suitable acquisition window can therefore improve sulfur selectivity. In this study， a high-sensitivity single-channel PFPD was designed and tested. The aim was to establish reproducible operating conditions for trace sulfur analysis. The detector consisted of five functional modules： the combustion and ignition module， the gas supply module， the optical collection and detection module， the temperature-control module， and the mechanical support and sealing module. The combustion zone consisted of a vertically mounted quartz chamber. The chamber had an outer diameter of 4 mm. Its inner diameter was 2 mm. Its total length was 17.5 mm. The effective combustion length was 12 mm. The calculated chamber volume was 37.68 mm^3^. Numerical simulation was used to examine flame propagation and self-extinction. A 12 mm effective length maintained a stable finger-shaped flame front. The flame was self-terminated near the lower limiting orifice at about 1.97 ms. The hydroxyl radical mass fraction decreased close to baseline within 5 ms， whereas the temperature decayed more gradually and approached its initial level at approximately 30 ms. These results supported the chamber design for periodic pulsed combustion. The optical unit used a quartz light guide and a BG-12 band-pass filter. The filter matched the sulfur emission band near 394 nm. A photomultiplier tube （PMT） recorded the time-resolved emission signal. Two electronic pneumatic control （EPC） units were used for gas regulation. One unit controlled hydrogen and air for the ignition chamber. The other unit controlled hydrogen and air for the combustion chamber. This design separated ignition conditions from analytical combustion conditions. It also reduced uncertainty caused by coupled gas adjustment. The optimization started with the ignition chamber. Then the combustion chamber was optimized under the fixed ignition baseline. Finally， detector temperature and PMT voltage were evaluated. Peak area， sensitivity， and signal-to-noise ratio （SNR） were used as indicators. Gas chromatographic tests used a sulfur-specific quartz capillary column. Nitrogen was used as the carrier gas. The gate delay was 6 ms. The gate width was 18 ms. The trigger level was 500 mV. A stable pulsed flame was obtained at an ignition-chamber hydrogen flow of 8.0 mL/min. The corresponding ignition-chamber air flow was 19.0 mL/min. Under this ignition baseline， the combustion chamber was further adjusted. A higher sulfur response was obtained at 5.0 mL/min hydrogen. The corresponding air flow was 3.7 mL/min. Detector temperature also affected the delayed sulfur emission. The response was low at 110-130 ℃. At about 150 ℃， the delayed sulfur signal was higher. Peak area and sulfur sensitivity were also higher at this temperature. Higher temperatures from 170 to 250 ℃ reduced the peak area and sensitivity. PMT voltage affected signal amplitude， SNR， and waveform fidelity. Increasing the voltage from 590 to 710 V improved the SNR. At 730 V， the delayed emission plateau showed peak clipping. This behavior indicated saturation or nonlinear response in the detection chain. Dynamic dilutions of a 10 μmol/mol hydrogen sulfide （H₂S） standard gas were used to evaluate the linear range. A 1.1 μmol/mol H₂S standard gas was used for the quantitative repeatability test and， after 30-fold dynamic dilution， for determination of the sulfur detection limit. The quantitative repeatability， evaluated from the peak areas of seven consecutive injections， was 0.37%. The sulfur detection limit was 4.1×10⁻^1^³ g/s， and the linear range spanned approximately two orders of magnitude. The developed PFPD showed stable pulsed combustion behavior. It also provided reliable quantitative response for trace sulfur detection. The dual-EPC design helped clarify the relationship between ignition and analytical combustion. The optimized conditions provide an experimental basis for self-developed PFPD systems.

含硫化合物（如H_2_S、羰基硫（carbonyl sulfide， COS）等）广泛存在于天然气、炼化过程气、工业尾气及恶臭排放等复杂基体中，其痕量检测与准确定量对过程安全、产品质量控制及环境排放监管具有重要意义^［1-5］^。火焰光度检测器（flame photometric detector， FPD）因对硫具有较高灵敏度而被广泛用于含硫化合物分析，但在复杂烃类基质中，其硫响应易受背景发光和火焰猝灭影响：烃类组分在火焰中燃烧生成的CO_2_会对硫检测产生干扰，高浓度烃类还可能引发火焰辐射猝灭，削弱硫特征发射信号。同时，火焰气体流动状态及待测分子结构差异也会改变FPD的响应行为，使其实测响应偏离理想的浓度平方关系^［6］^。

为减弱烃类干扰并提高硫检测灵敏度，研究人员在常规FPD基础上发展出了脉冲式火焰光度检测器（pulsed flame photometric detector， PFPD）。与FPD相比，PFPD利用硫发光与烃类背景在时间域上的差异实现选择性检测，但其性能并非仅由时间门控参数决定。延迟发光窗口的判别、脉冲火焰的稳定形成，以及热边界条件和探测增益链路的匹配，均会影响时间域波形、信噪比（SNR）和定量重复性。

针对上述问题，已有研究从发光机理、检测原理及应用拓展等方面开展了探索。Syty等^［7］^和Fair等^［8］^对富燃火焰中硫相关发光物种的生成及辐射跃迁过程进行了研究，为延迟发光的时间分辨判别提供了理论基础；Amirav等^［9-11］^提出并发展了PFPD，通过周期性脉冲燃烧与时间分辨采集实现硫、磷发光与烃类背景的时间域分离，从原理上提升了检测选择性；相关研究^［12-14］^还围绕硫发光猝灭、光学收集效率及探测链路优化展开，为仪器稳定运行和实际样品适配提供了参考。在应用方面，Massimi等^［15］^在热脱附-气相色谱（thermal desorption-gas chromatography， TD-GC）平台上实现了质谱（mass spectrometry，MS）、火焰离子化检测器（flame ionization detector， FID）和PFPD的多检测器并行采集。Torres-Herrera等^［16］^则通过优化进样器温度和载气流速改善了GC-PFPD对含硫组分的定量响应。此外，5383 PFPD的公开技术资料显示^［17］^，燃烧室内径、空气流量和时间门控参数均会影响元素检测条件的建立。其中，燃烧室内径主要影响脉冲燃烧空间和发光持续时间，空气流量影响脉冲频率和火焰重复性，门延迟及门宽决定延迟发光信号的采集区间。这表明，现有PFPD的性能提升已不再局限于单一门控参数，而是依赖于供气组织、燃烧室结构和检测链路的协同优化。总体来看，已有研究从发光机理、时间门控和典型样品检测等方面推动了PFPD的发展，为其选择性检测和应用拓展提供了重要基础。然而，相关工作主要围绕特定样品或特定应用场景的方法建立展开，工作条件的确定多依赖经验调节，针对PFPD中引火室气氛与燃烧室气氛相互影响的研究仍较少。尤其是，如何通过供气参数解耦调控、关键运行参数逐级筛选和推荐工作条件建立，形成面向仪器研制与性能评价的可复现实验流程，仍有待进一步明确。

基于此，本文以PFPD稳定脉冲燃烧和痕量硫定量检测为目标，采用双路电子压力控制（electronic pneumatic control， EPC）实现引火室与燃烧室气路参数的解耦调控，构建“点火基准建立-燃烧室供气参数优化-检测温度与信号增益匹配”的优化路线。通过系统考察燃气流量及配比、检测器温度和光电倍增管（photomultiplier tube， PMT）工作电压等关键参数，并在统一延迟发光积分窗口下对峰面积、灵敏度和信噪比进行定量表征，建立具有重复性、可解释性和工程复现性的推荐工作条件，为PFPD的结构设计、运行参数优化、硫延迟发光响应及性能测试提供实验依据和方法学参考。

## 1 PFPD结构设计与仿真

本研究自主研制的单通道PFPD，结构示意见图1。检测器由燃烧与点火模块、气路模块、光学采集与检测模块、温度控制模块以及机械支撑与密封模块组成。在此结构基础上，采用数值模拟考察小体积燃烧室内火焰传播及自熄灭过程，并分析检测器的脉冲燃烧机理。其目标是在小体积燃烧空间内实现稳定脉冲燃烧，并将延迟发光信号高效、低背景地传输至PMT进行时间分辨检测。

**图1:**
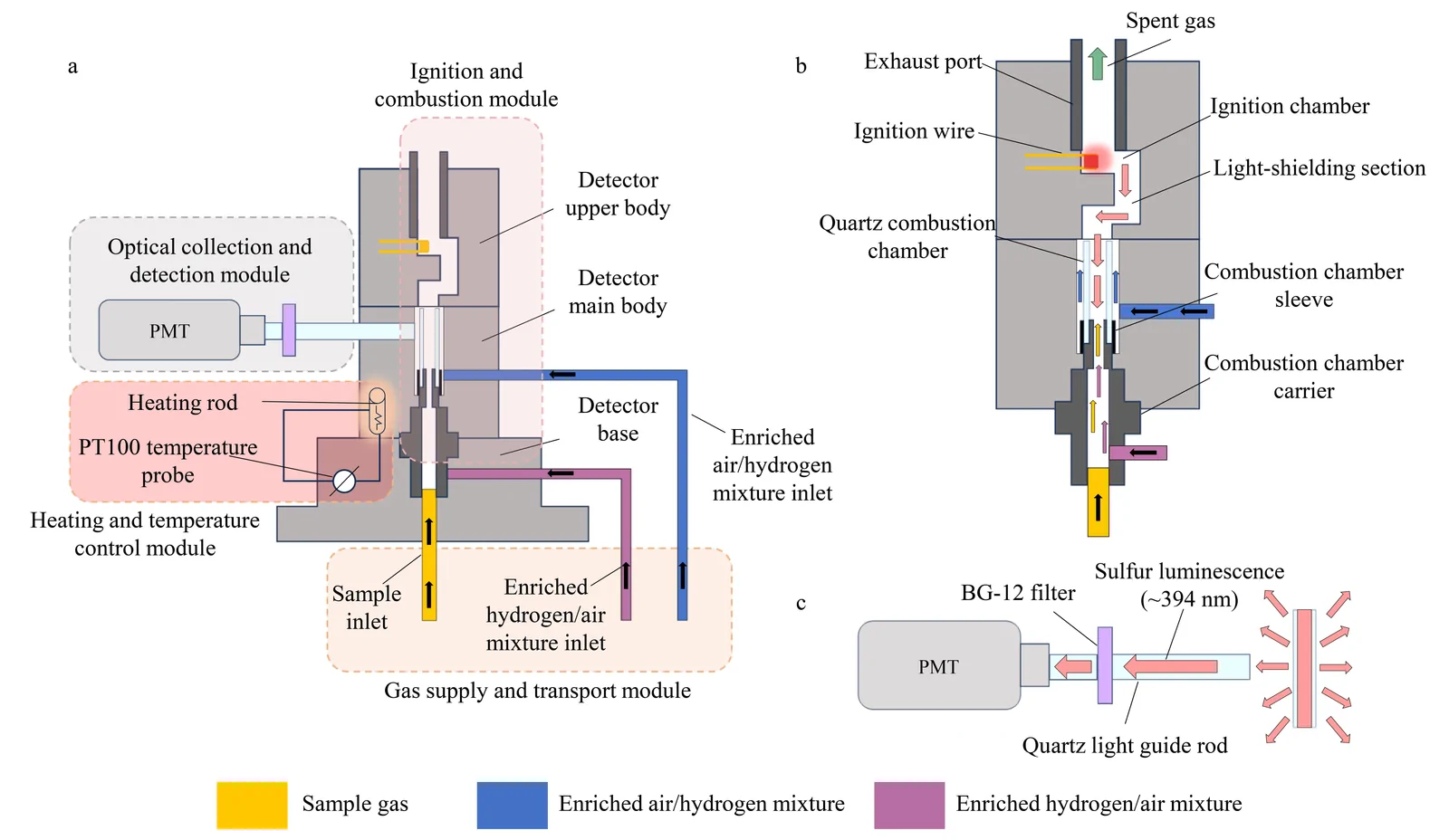
PFPD的结构组成及火焰传播和光学检测路径示意图

### 1.1 燃烧与点火模块设计

燃烧与点火模块是检测器实现脉冲燃烧和时间分辨检测的核心结构，其在检测器中的位置见图1a。检测器采用竖直安装的小体积石英燃烧室作为主要燃烧区域。燃烧室材料为高纯石英，外径4 mm，内径2 mm，总长度17.5 mm，有效燃烧长度12 mm，体积约为37.68 mm³。该尺寸设计主要基于硫检测灵敏度与脉冲燃烧稳定性的综合权衡。研究表明^［17］^，硫检测宜采用内径为2 mm的燃烧室，其硫延迟发光时间窗口较3 mm更长，更有利于提高硫通道灵敏度。为进一步确定燃烧室有效长度（*L*），在保持燃烧室内径为2 mm的条件下，选取燃烧室H_2_流量2.0 mL/min、空气流量3.5 mL/min作为代表性工况，对不同长度结构进行了数值比较，结果见图2。图2a显示，*L*显著影响火焰前锋形态的稳定性，仅当*L*=12 mm时，火焰可全程维持稳定的手指状前锋，过短或过长的燃烧室均会诱发郁金香形变，并导致信号展宽或波形振荡。图2b进一步给出了燃烧室轴线中点处羟基自由基（·OH）质量分数的瞬态响应。其中，*L*=12 mm时，·OH峰值最高，表明该长度下中部区域的主燃烧反应活性较强。结合图2a中*L*=12 mm条件下较稳定的火焰前锋形态可知，该长度在火焰传播稳定性和中部反应强度之间具有较好的匹配关系。

**图2:**
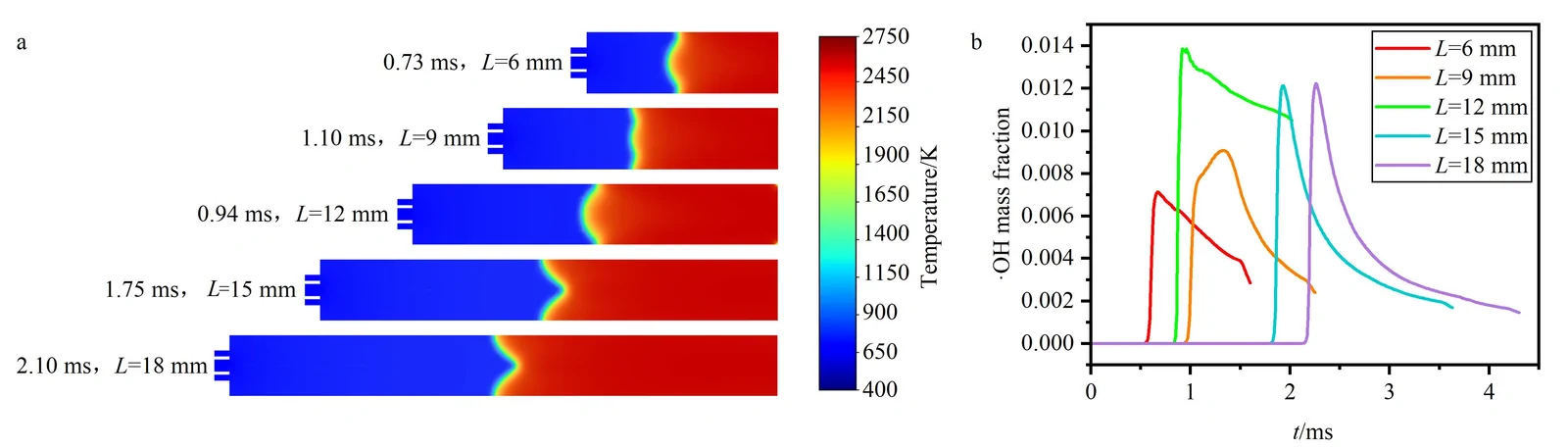
不同燃烧室有效长度（*L*）下火焰前锋形态及·OH响应特征比较

因此，本文最终选取内径2 mm、有效燃烧长度12 mm的小体积石英燃烧室作为较优结构参数。燃烧室下端通过燃烧室承载器定位安装，承载器顶部设置孔径为1 mm的限流通孔，用于限制火焰继续向下传播并形成自熄灭条件，从而维持脉冲燃烧过程的周期性和稳定性。

如图1b所示，点火模块位于燃烧室上方的引火室内，采用持续加热式点火丝实现周期性点燃。引火室体积约为154.85 mm³，与燃烧室体积之比约为4∶1，有利于形成相对稳定的引燃环境，并为前一周期燃烧产物排出及下一周期新鲜混合气补充提供空间。点火丝采用镍铬合金材料，丝径0.3 mm，工作电压6.28 V。与脉冲放电点火相比，持续加热式点火结构更简单、触发更稳定，更适用于小体积燃烧室条件下的重复脉冲燃烧。为减弱点火丝红热辐射对检测信号的直接干扰，引火室与石英燃烧室之间设置遮光隔离段（light-shielding section），内径3 mm，长度20 mm（见图1b）。该结构一方面为火焰由引火室向燃烧室传播提供通道，另一方面可阻挡点火丝红热辐射直接进入侧向光学检测路径，从而减小点火区杂散发光对硫延迟发光信号采集的干扰。

### 1.2 气路设计

现有商业化PFPD通常涉及燃烧气、空气及辅助气等多个供气参数的协同调节^［17］^。在这种调节方式下，改变某一路气体流量往往会同时影响点火稳定性、火焰传播状态、脉冲频率及延迟发光强度，使点火条件与分析燃烧条件难以独立优化。

针对这一问题，本研究采用双路EPC分别控制引火室辅助气路和燃烧室中心气路，实现点火过程与分析燃烧过程在供气层面的解耦。PFPD气路示意图见图3。其中，中心气路用于将色谱柱流出的待测组分直接送入燃烧室内部，并在该区域完成主要燃烧和延迟发光；辅助气路则主要用于置换前一周期的燃烧产物、重建引火室气氛并参与下一轮火焰触发。通过独立调节两路气流的流量及氢气和空气比，可调控燃烧室内外气体组成及混合状态，进而调节火焰传播节律，使系统在避免连续火焰形成的同时保持较好的脉冲重复性和发光强度。为此，检测器在结构上设置了两路独立进气接口，分别对应中心气路和辅助气路，接口直径均为2 mm，并由外部流量控制单元实现独立调节。该设计有助于减少中心气流与辅助气流在检测器内部动态混合过程带来的不确定性，并为后续运行窗口优化提供结构基础。

**图3:**
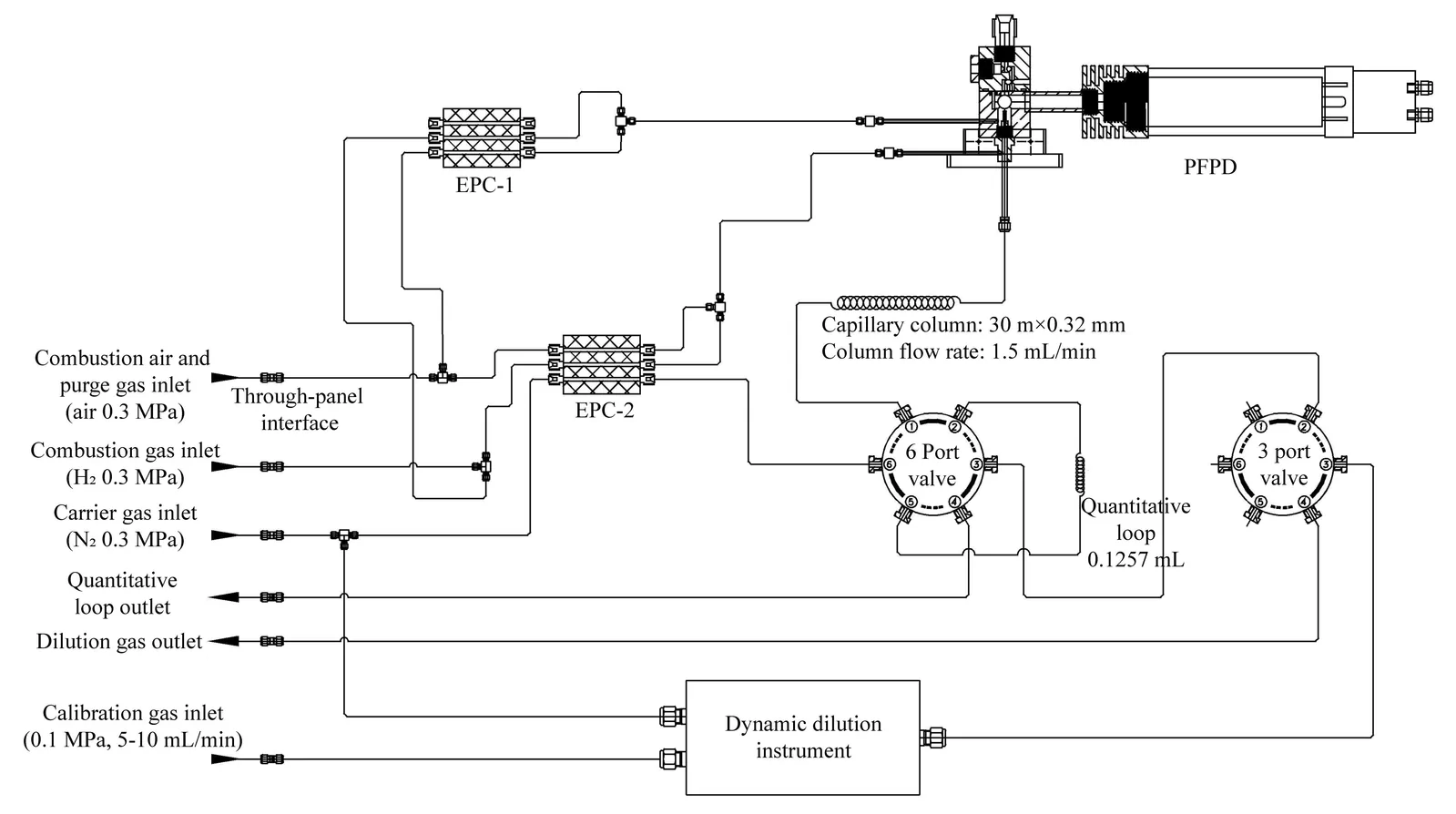
PFPD气路图

### 1.3 光学采集与检测模块设计

光学采集与检测模块沿燃烧室侧向布置（见图1a），用于收集脉冲燃烧产生的延迟发光并完成光电转换（见图1c）。燃烧发光首先由侧向设置的石英导光棒收集，再依次传输至滤光片和PMT。导光棒采用石英材料，直径为8 mm，长度为50 mm，一端靠近燃烧区布置，另一端与滤光片及PMT输入端对接。本文选用的滤光片为BG-12带通滤光片，其主透过波段位于390~410 nm，在该波段具有较高透过率，可有效透过约394 nm的硫特征发光峰，同时抑制其他波长的背景辐射。该结构可在保证采光效率的同时，将高温燃烧区与PMT在空间上分离，从而减弱热辐射和热传导对PMT工作的影响。

### 1.4 温度控制模块设计

温度控制模块用于维持检测器主体及燃烧区周围的稳定温度，以避免水汽冷凝，减少高沸点或活性组分在壁面上的吸附滞留，从而提高脉冲燃烧的重复性。考虑到PFPD工作过程中温度过低易引起冷凝和记忆效应，而温度过高又可能影响火焰时域特征，因此检测器温度需控制在合适范围内。如图1a所示，检测器采用加热棒进行恒温加热，由PT100探头实时监测温度并反馈控制。为减小检测器不同部位之间的温度差异，检测器外部设置隔热罩，以降低局部温度波动导致的气体密度变化、冷凝及信号漂移，从而提高了不同温度设定下实验的稳定性和重复性。

### 1.5 机械支撑与密封

检测器上壳、检测器主体和检测器底座共同构成检测器的机械支撑框架，主要金属部件采用316不锈钢制造，以满足加热工作条件下对机械强度、耐温性和耐腐蚀性的要求。检测器整体采用分体式装配结构，便于部件调节、拆卸维护和清洗。

燃烧室通过承载器定位安装，以减小石英构件装配过程中的机械应力并保证其在燃烧区内的稳定位置；导光棒采用侧向固定方式，以维持与燃烧发光区域之间稳定的光学耦合关系；进气接口采用螺纹式密封接头连接，以兼顾气密性和重复拆装的便利性。考虑到PFPD对燃烧室壁面状态和内部洁净度较为敏感，仪器结构设计中兼顾了燃烧室快速更换、主体便于清洗及采光通道不易污染等要求，从而提高了检测器长期运行的稳定性和重复性。

### 1.6 火焰传播与自熄灭仿真

火焰能否沿燃烧室稳定传播并在预定位置可靠终止，是小体积PFPD实现周期性脉冲燃烧的关键，其周期性工作原理如图4所示。在1.1节完成燃烧室几何参数筛选的基础上，为进一步验证所设计燃烧室及承载器限流结构的合理性，本文对火焰传播与自熄灭过程进行了数值模拟。

**图4:**
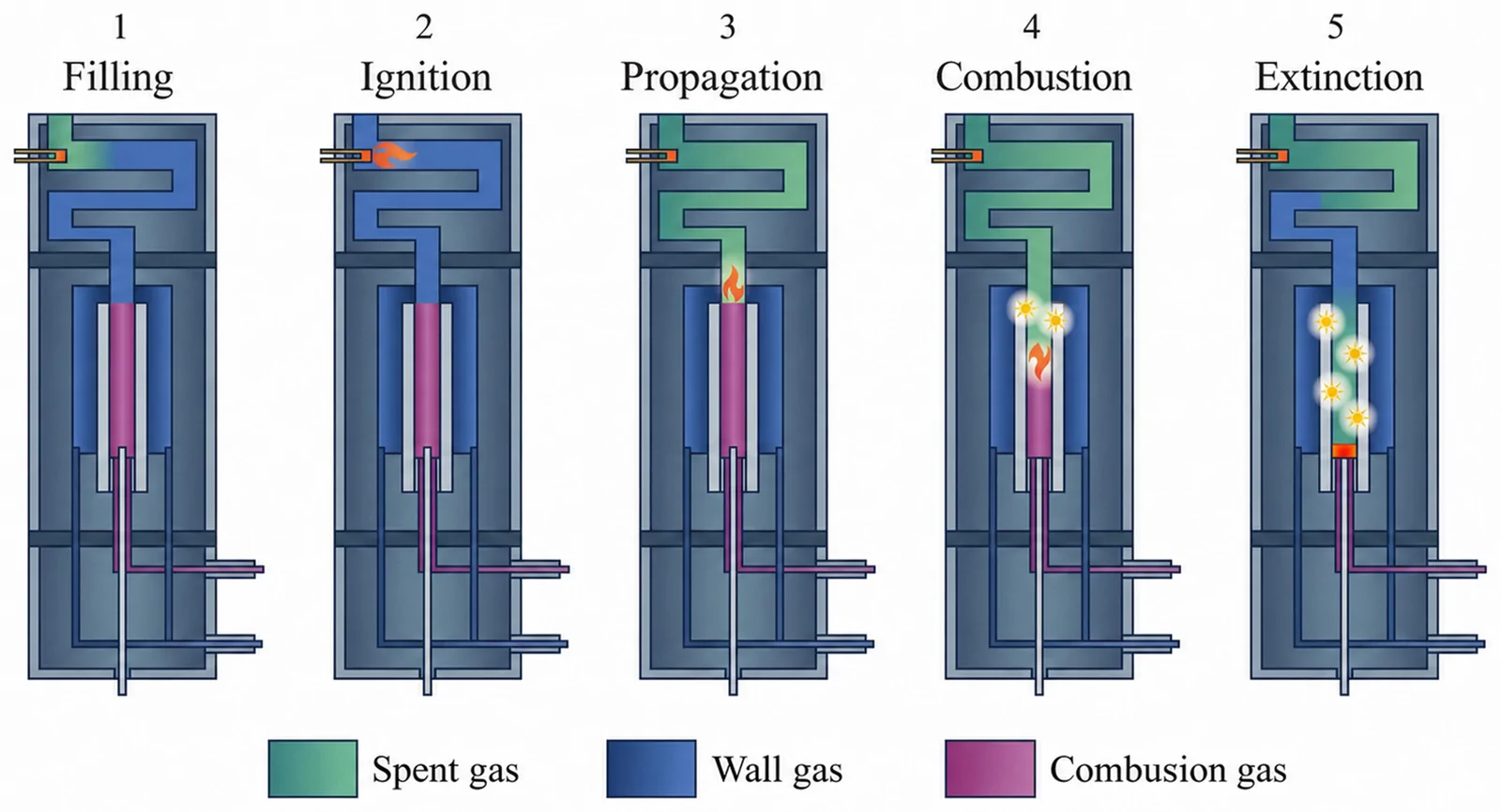
PFPD工作原理

仿真条件与1.1节用于结构筛选的代表性工况一致，即以燃烧室H_2_流量2.0 mL/min，空气流量3.5 mL/min作为代表性工况，考察火焰在内径2 mm、有效长度12 mm的石英燃烧室内由上向下传播的过程。仿真结果见图5。结果表明，点火后火焰可沿燃烧室轴向向下延烧，并在传播至底部限流区域附近时因局部几何限域、热损失增强及流动条件变化而停止推进，1.97 ms时发生自熄灭，标志着火焰主链式燃烧反应在2 ms内已基本完成，说明承载器顶部限流结构能够为火焰提供明确的终止边界。

**图5:**
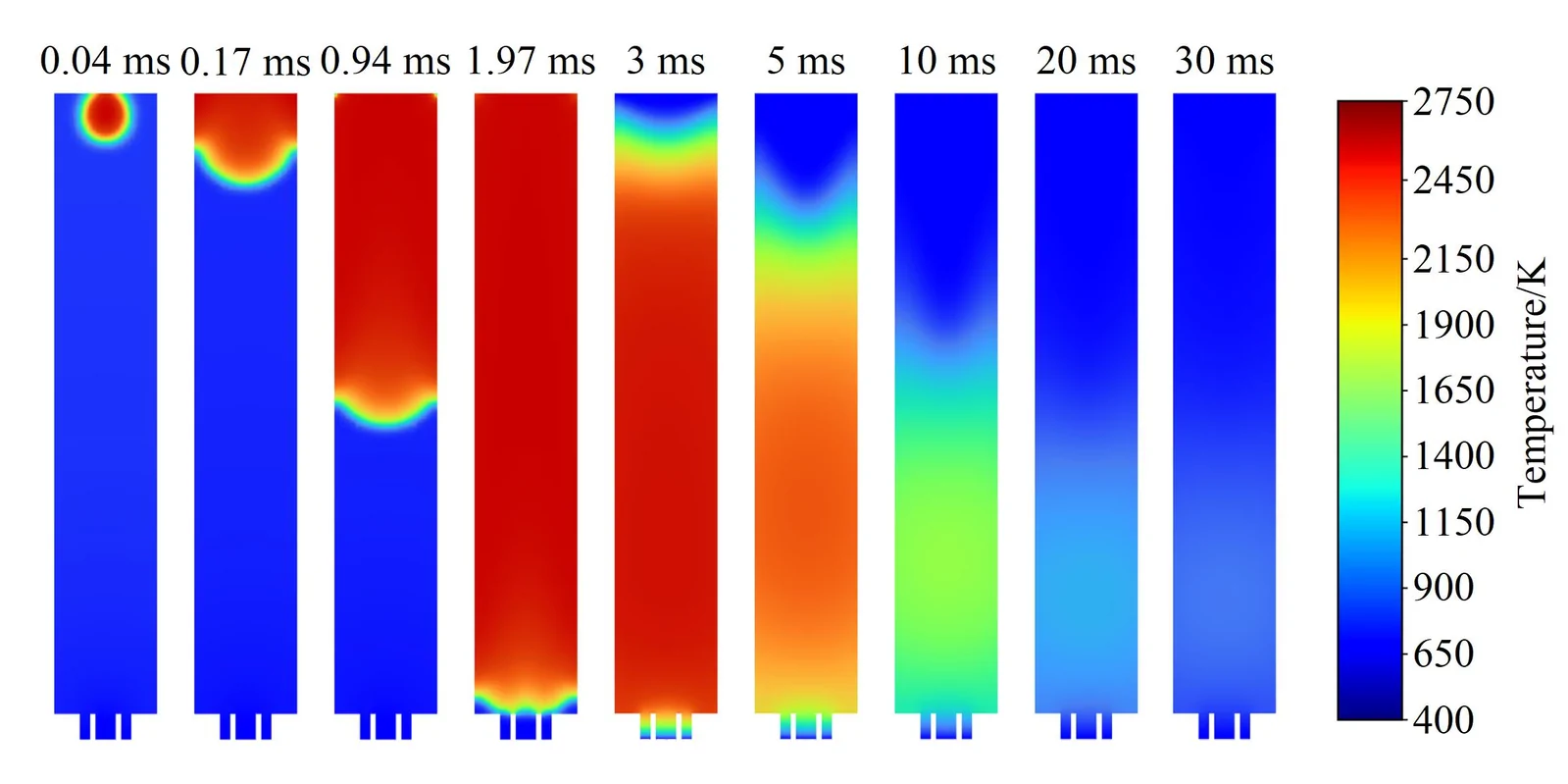
燃烧室内火焰传播与自熄灭过程的温度场仿真结果

为进一步表征该过程的时域衰减特征，在燃烧室轴线中点设置监测点，提取表征火焰主链式反应的·OH自由基质量分数与温度的瞬态变化（见图6）。结果显示，火焰前锋经过监测点时，两者迅速升高；在火焰自熄灭后，·OH质量分数迅速下降，并在5 ms内衰减至接近基线水平，表明与主燃烧反应相关的活性自由基已基本消耗完毕。相比之下，体系温度下降较为缓慢，主火焰消失后燃烧室内仍维持较高温度，表明其进入无明显主火焰传播的高温余焰反应阶段。该结果表明，所设计燃烧结构能够较好地实现火焰传播与自熄灭过程，为检测器稳定脉冲运行提供了结构基础，同时为硫与烃类发光的时间域分离提供了关键的反应动力学边界条件。

**图6:**
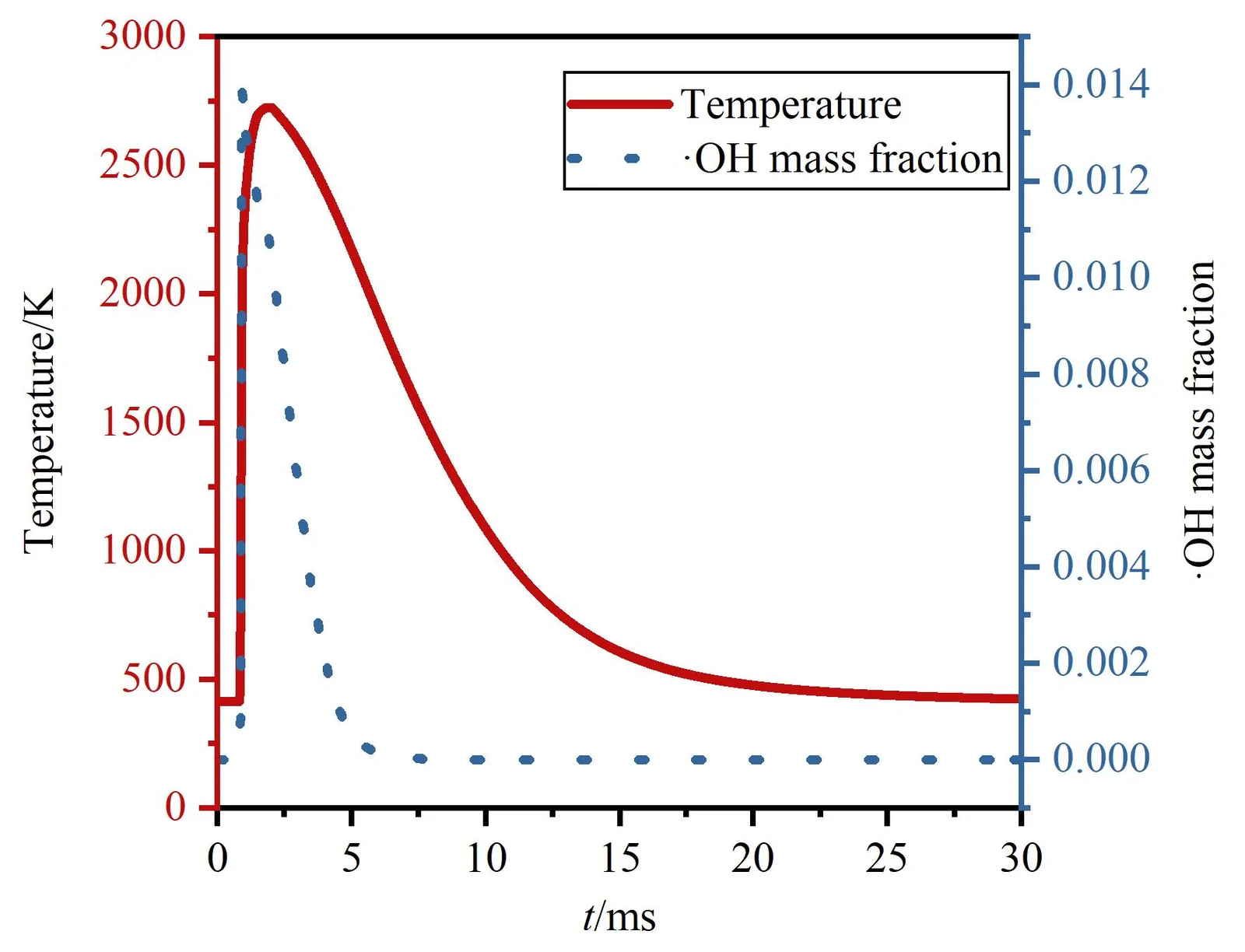
监测点处·OH质量分数与温度的瞬态响应曲线

## 2 PFPD运行参数优化

### 2.1 气体流量与配比优化

PFPD的脉冲燃烧及硫通道响应对气氛条件高度敏感。气体总流量及氢气和空气比会影响火焰的形成、传播和能量释放过程，进而改变脉冲频率、燃烧稳定性以及硫延迟发光强度。当气氛条件偏离合适范围时，易出现脉冲频率下降或燃烧不稳定等现象，从而降低检测的重复性和灵敏度。典型表现之一为Tick-Tock模式，即有效发光脉冲间歇出现，通常与燃烧产物置换不足及火焰传播不稳定有关^［17］^。

为系统考察气氛条件对脉冲火焰和硫响应的影响，本研究采用双路EPC将引火室与燃烧室气路解耦，分别调控点火稳定性和主燃烧区气氛条件。评价指标选取硫信号峰面积和硫灵敏度，其中灵敏度按标准动态法计算^［18］^，并以注入硫质量进行归一化。基于此，后续将首先讨论引火室气氛对点火及火焰核稳定性的影响，再分析燃烧室气氛对硫延迟发光响应的作用。

#### 2.1.1 引火室独立点燃实验

为建立稳定的点火基准，在燃烧室气体关闭条件下，考察引火室H_2_与空气流量对点火稳定性及脉冲频率的影响。以脉冲是否连续产生、波形是否一致作为稳定性判据，并以实际燃烧频率（*f*）作为量化指标，实验条件见表1。

**表1:** 引火室独立点燃实验条件

Parameter	Value
Combustion chamber H_2_ flow/（mL/min）	0
Combustion chamber air flow/（mL/min）	0
Combustion chamber N_2_ flow/（mL/min）	1.5
Ignition chamber H_2_ flow/（mL/min）	0
Ignition chamber air flow/（mL/min）	16.0
PMT voltage/V	630
Trigger level/mV	500
Ignition voltage/V	6.28
Detector temperature/℃	150

在引火室空气流量16.0 mL/min条件下，逐步提高H_2_流量。不同引火室H_2_流量条件下燃烧脉冲的时间波形见图7。当H_2_流量≤4.0 mL/min时未形成稳定点火（*f*=0 Hz），表明此时混合气接近或低于可燃下限；当H_2_流量增至6.0 mL/min后出现规则脉冲（*f*=2.70 Hz），说明系统已进入可自持燃烧区；当H_2_流量8.0 mL/min时，脉冲频率达到最大（*f*=3.03 Hz），且波形重复性最好；继续增至10.0 mL/min时，频率略降至2.86 Hz，并出现轻微波形畸变，说明偏富燃条件下氧供给不足，不利于脉冲燃烧稳定维持。由此可见，在空气流量16.0 mL/min条件下，引火室H_2_流量在6.0~8.0 mL/min范围内可实现稳定脉冲燃烧，其中8.0 mL/min为较优值。

**图7:**
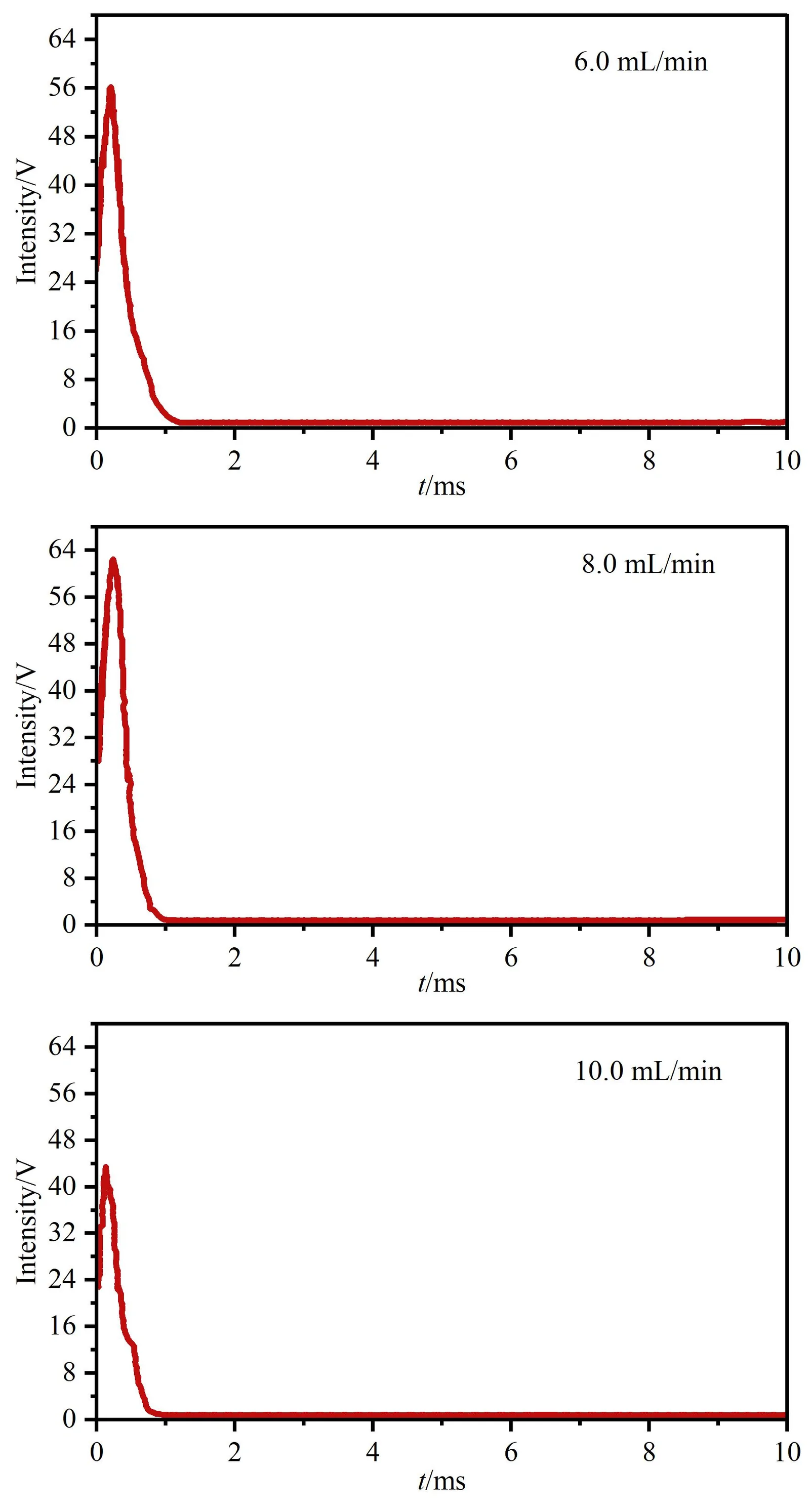
不同引火室H_2_流量条件下燃烧脉冲的时间波形

在此基础上，固定引火室H_2_流量为8.0 mL/min，进一步提高空气流量，结果见表2。随着空气流量由13.0 mL/min增至19.0 mL/min，氢气和空气比由0.62降至0.42，脉冲频率持续升高。当空气流量<15.0 mL/min时，脉冲频率较低且波形稳定性较差，说明氧供给不足限制了燃烧反应的充分进行；随着空气流量增加，脉冲逐渐稳定，并在19.0 mL/min附近达到本实验考察范围内的最高频率。空气流量为19.0 mL/min时对应的氢气和空气比为0.42，与H_2_在空气中化学计量燃烧的配比一致，表明此时引火室气氛更有利于实现稳定、充分的点火过程。需要说明的是，受本实验所用EPC流量控制范围限制，气体流量上限为19.0 mL/min，因此未继续测试更高空气流量。

**表2:** 固定引火室H_2_流量（8.0 mL/min）并逐步提高引火室空气流量时的气体配比与燃烧频率

Ignition chamber air flow/（mL/min）	Ignition chamber H_2_/Air	Combustion frequency/Hz
13	0.62	0.64
14	0.57	2.08
15	0.53	2.56
16	0.50	3.03
17	0.47	3.23
18	0.44	3.57
19	0.42	3.85

Combustion chamber H_2_ flow： 0 mL/min； combustion chamber air flow： 0 mL/min.

综合频率和波形稳定性，引火室独立点燃实验的优选流量组合为H_2_流量为8.0 mL/min、空气流量为19.0 mL/min。后续燃烧室供气参数优化及硫检测实验均采用该参数作为引火基准。

#### 2.1.2 燃烧室-引火室联合燃烧实验

在引火室点火条件固定H_2_流量为8.0 mL/min、空气流量为19.0 mL/min的基础上，接通燃烧室气路，考察火焰向下传播及主燃烧区构建对燃烧室气氛的依赖。实验中固定燃烧室空气流量为3.5 mL/min，逐步提高燃烧室H_2_流量，并以实际燃烧频率*f*和单脉冲波形作为评价指标，结果见表3。

**表3:** 固定燃烧室空气流量（3.5 mL/min）并逐步提高燃烧室H_2_流量时的气体配比与燃烧频率

Combustion chamber H_2_ /（mL/min）	Combustion chamber H_2_/Air	Combustion frequency/Hz
0	0	4.00
1.0	0.29	4.17
2.0	0.57	4.17
3.0	0.86	4.35
4.0	1.14	4.35
5.0	1.43	4.76

Ignition chamber H_2 _flow： 8.0 mL/min； ignition chamber air flow： 19.0 mL/min.

随着燃烧室H_2_流量增加，燃烧频率由4.00 Hz缓慢升高至4.76 Hz，但脉冲波形由“双峰”逐渐演变为“单峰”，见图8。当燃烧室H_2_流量为0 mL/min时，发光主要集中于引火室及燃烧室上部，PMT波形表现为窄而高的初始峰；当H_2_增至1.0~2.0 mL/min后，燃烧室中下部逐渐形成可燃混合气，火焰开始向下传播，并在PMT视场内建立新的体积燃烧区，从而在约1~6 ms区间出现明显第二峰。随着燃烧室H_2_进一步增加，在空气固定条件下混合气逐渐趋于富燃，第二峰峰高及积分面积反而减小，表明主燃烧区有效发光受到抑制。这可能与氧供给不足及总流量增加导致停留时间缩短有关。

**图8:**
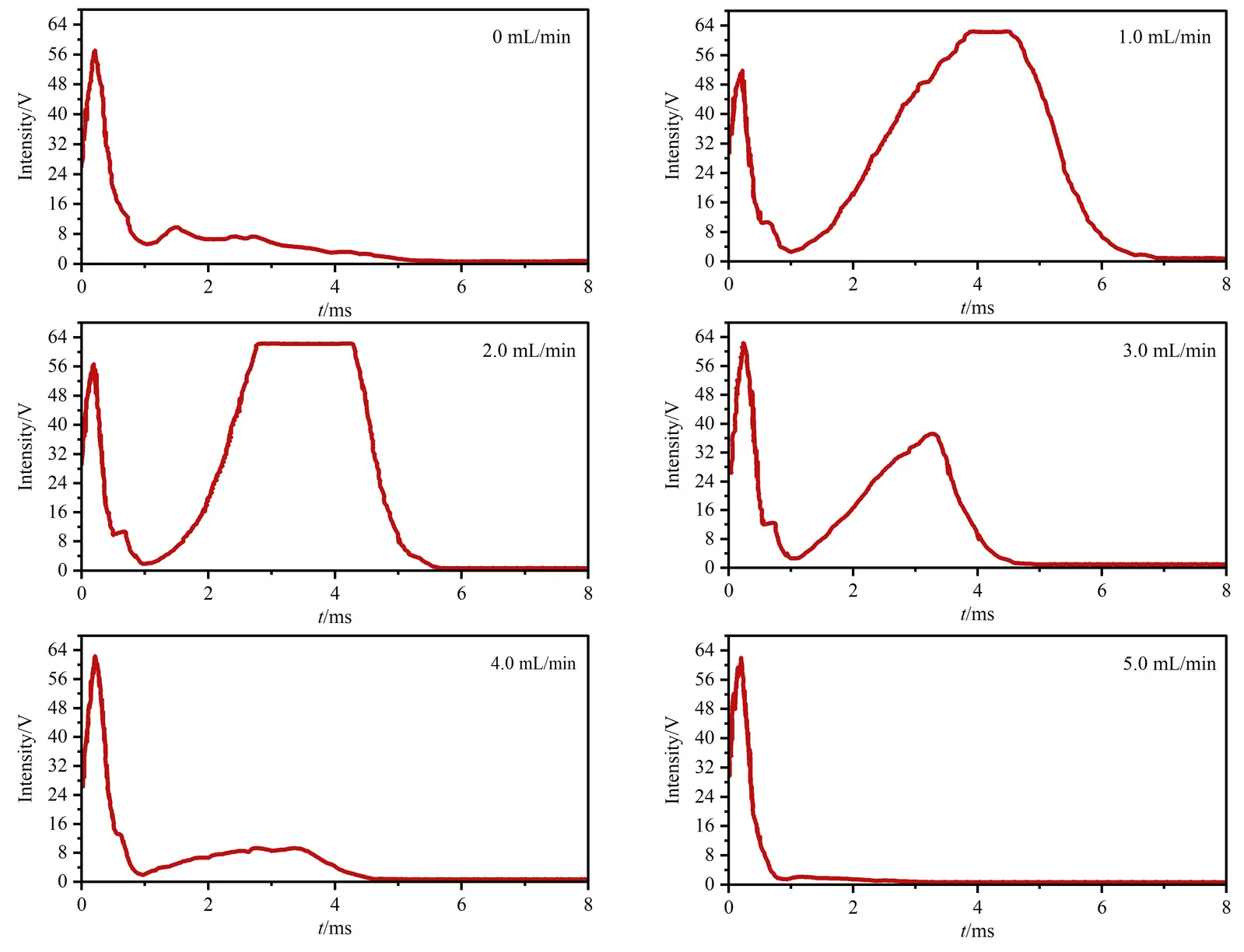
不同燃烧室H_2_流量条件下燃烧脉冲的时间波形

当燃烧室H_2_流量为5.0 mL/min、空气流量为3.5 mL/min时，硫通道延迟发光平台明显减弱并发生时间偏移，见图9，表明该条件下燃烧室可能处于氧受限状态。为此，在保持燃烧室H_2_流量为5.0 mL/min和引火室参数不变的条件下，进一步扫描燃烧室空气流量，结果（见图10和图11）表明，燃烧室空气对硫通道响应具有明显阈值效应：当空气流量由3.4 mL/min增至3.6 mL/min时，峰面积和信噪比均显著提高；当空气流量为3.7 mL/min时，峰面积、信噪比和灵敏度分别达到99 405 μV·s、212.7和24.81 μV·s/（pg S），为最优条件；继续增大至3.8~4.0 mL/min后，上述指标有所下降。综合来看，燃烧室空气流量适宜范围为3.6~4.0 mL/min，因此选取3.7 mL/min作为后续实验条件；空气流量不高于3.5 mL/min时，硫响应较弱，不作为推荐条件。

**图9:**
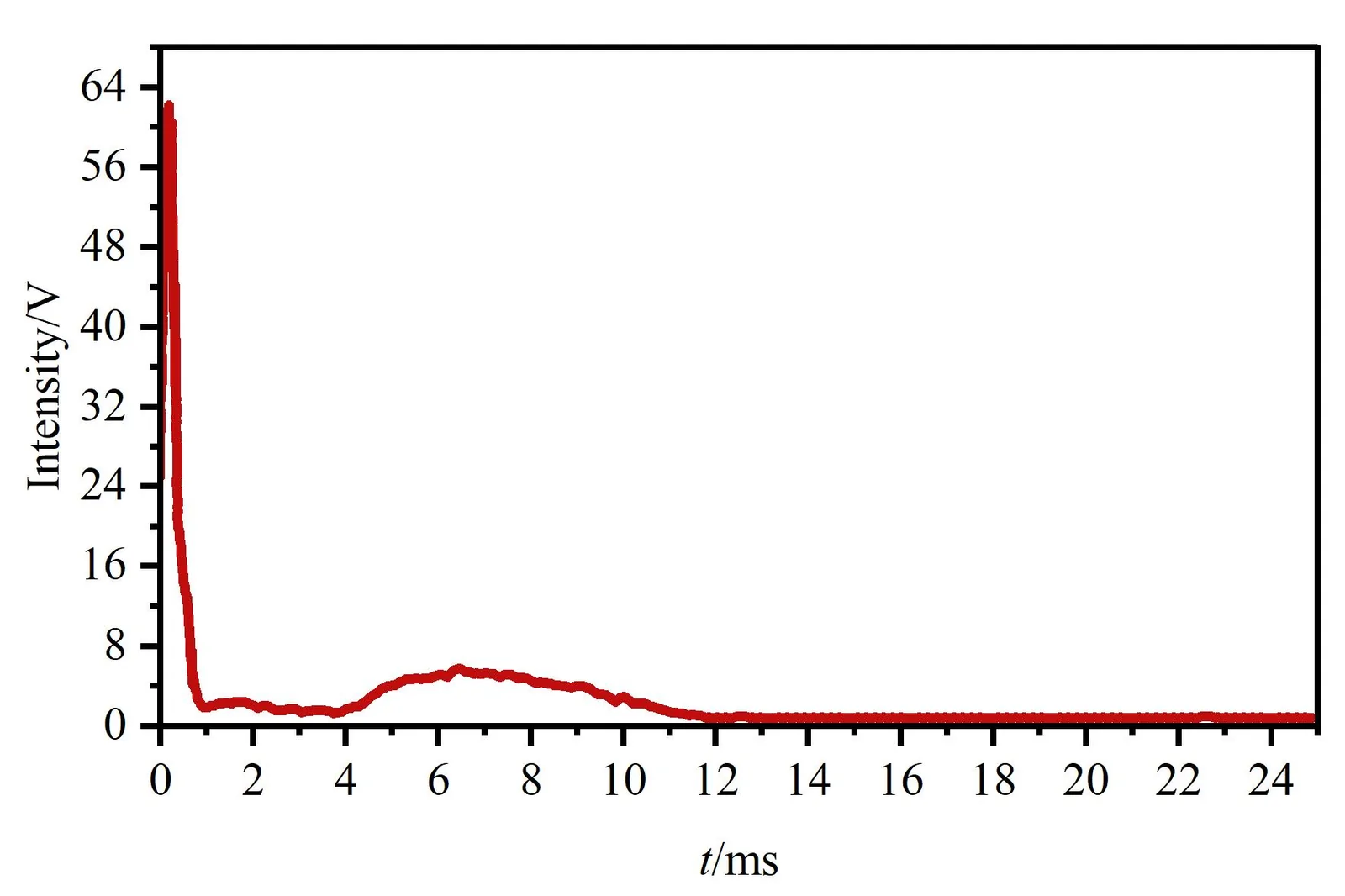
氧气不足工况下硫发光响应的时间波形

**图10:**
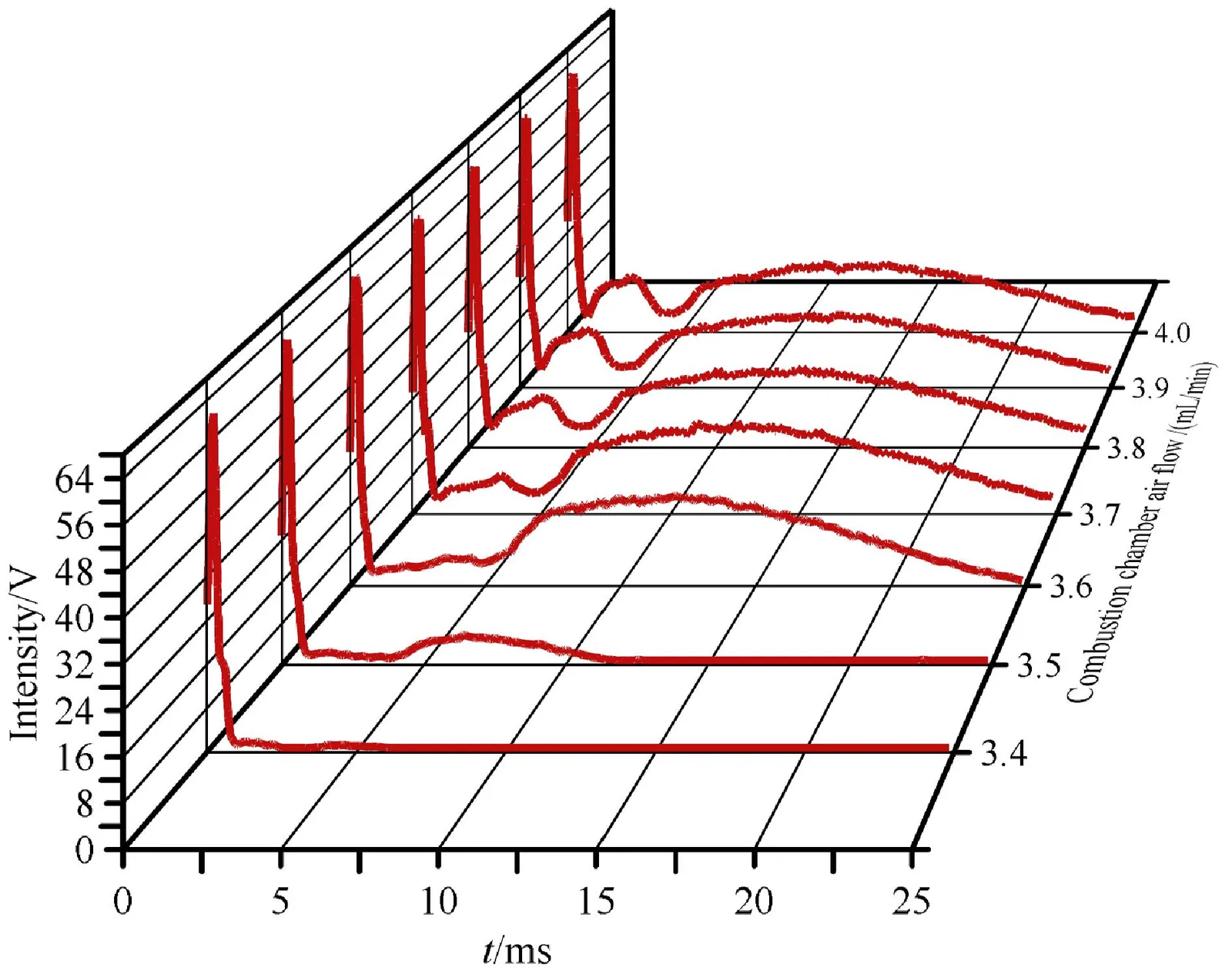
不同燃烧室空气流量下硫发光响应三维叠加图

**图11:**
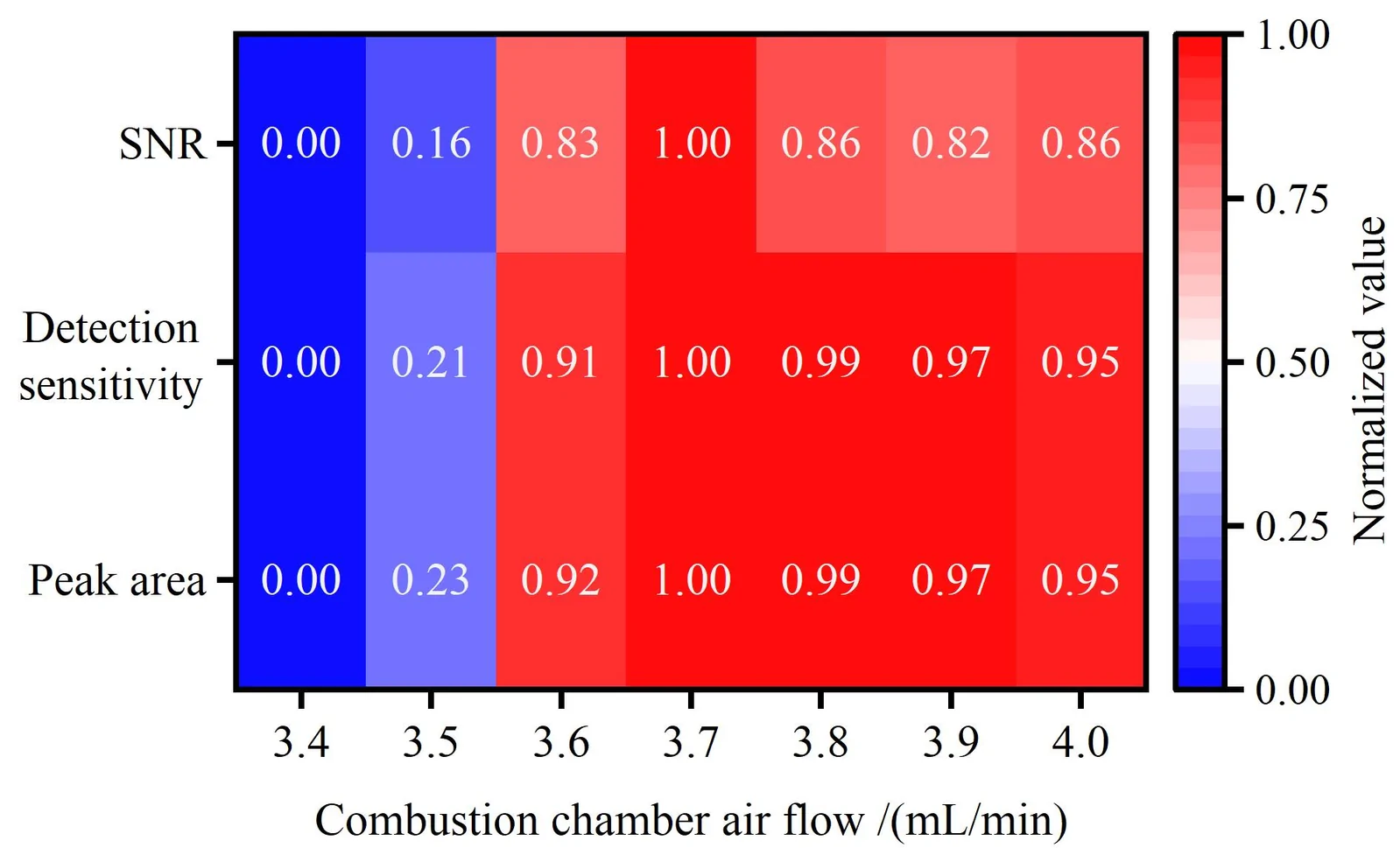
峰面积、检测灵敏度与SNR随燃烧室空气流量的热力图（数据已归一化）

### 2.2 检测器温度优化

检测器温度决定了燃烧室及余焰区的热边界条件，并影响含硫中间体的生成、转化和失活过程，从而改变硫延迟发光平台及积分响应。在上述已优化气体流量、配比和点火参数条件下，对检测器温度在110~250 ℃范围内进行梯度扫描。各温度点达到热平衡后采集硫通道时间波形，并在统一延迟积分窗口内计算峰面积和信噪比，结果见图12和图13。

**图12:**
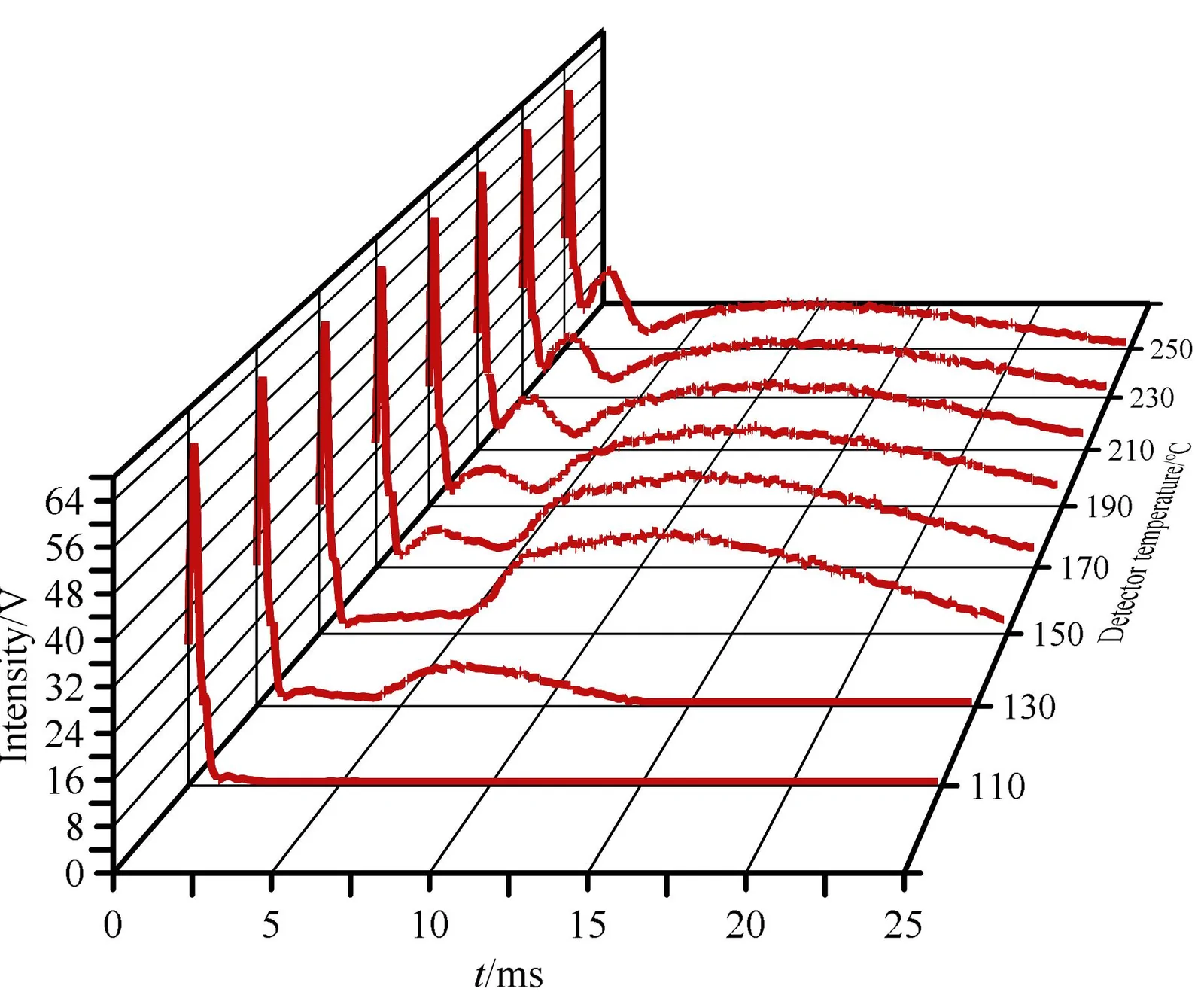
不同检测器温度下硫发光响应三维叠加图

**图13:**
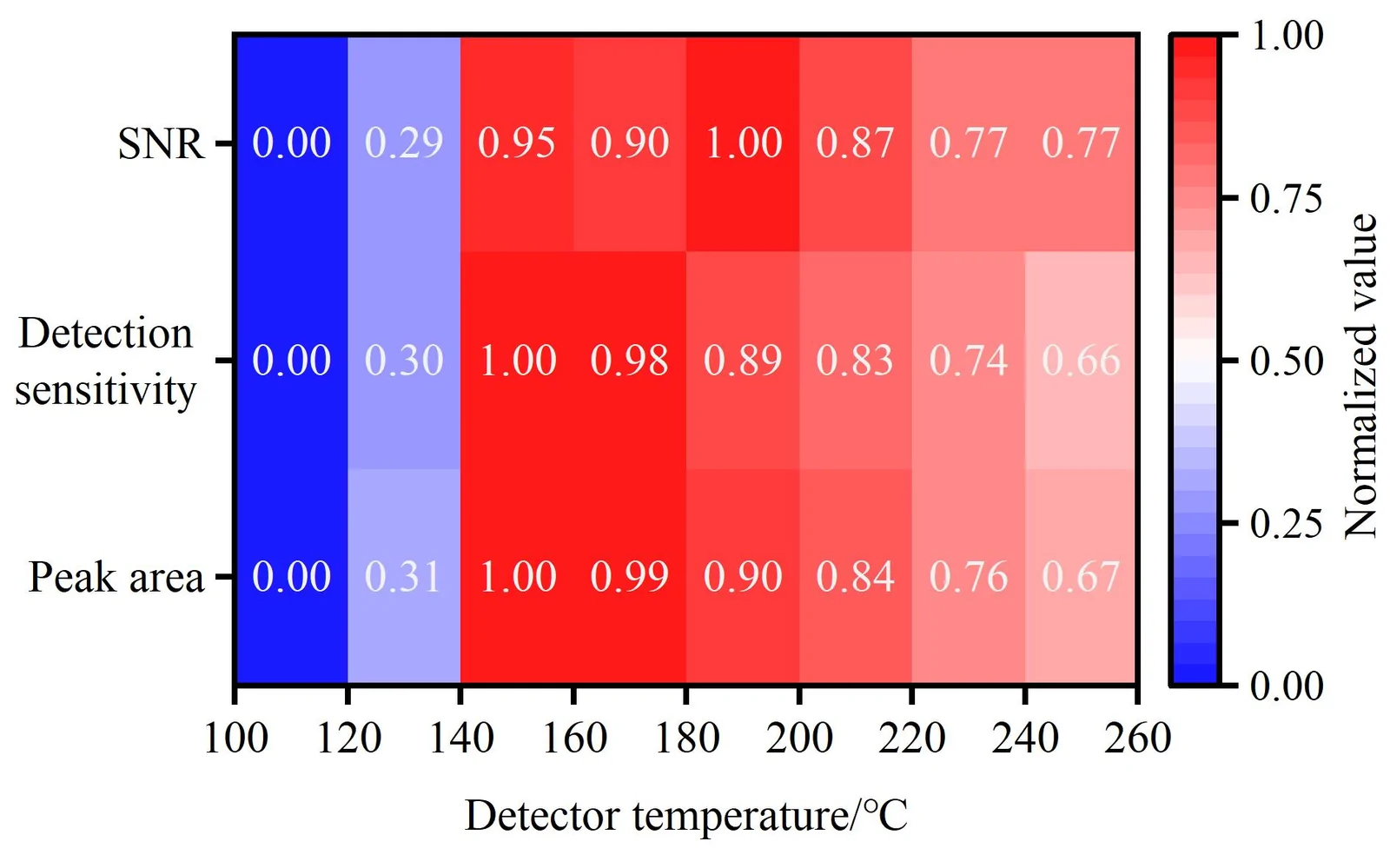
峰面积、检测灵敏度与SNR随温度变化的热力图（数据已归一化）

结果表明，检测器温度对硫延迟发光波形和定量响应均有影响。在110~130 ℃时，延迟发光平台难以建立，信号强度较低；当温度升至150 ℃左右时，延迟发光平台较为稳定，峰面积和硫灵敏度达到最大；继续升温至170~250 ℃后，延迟平台仍可观察到，但峰面积和灵敏度下降，并伴随波形前缘增强和平台展宽。上述结果表明，检测器温度会影响硫延迟发光信号的形成和采集，150 ℃左右可作为硫通道检测的推荐工作温度。

### 2.3 PMT工作电压优化

在完成气体流量优化并确定检测器温度工作窗口后，PMT工作电压成为进一步影响硫通道检测性能的关键参数。PMT电压决定信号倍增增益，进而影响峰高和峰面积，也会同步放大噪声，在高电压下可能引起探测链路的非线性响应。因此，在其余运行参数保持不变的条件下，对590~730 V范围内的PMT工作电压进行阶梯扫描，以确定兼顾响应强度、信噪比和峰形完整性的工作区间。

结果表明，在590~730 V范围内，随着PMT电压升高，硫通道时间响应幅值逐渐增大，瞬时脉冲峰和延迟发光平台均呈增强趋势，见图14。峰高随电压逐步增加，信噪比在590~710 V区间内亦随电压升高而改善，说明此阶段信号增益的提升超过了噪声放大的幅度。当电压升至730 V时，峰高仍继续增加，但信噪比明显下降，同时延迟发光平台顶部出现“削顶”现象，表明探测链路已接近或进入饱和状态，导致有效信号信息被压缩，从而使检测性能下降，见图15。

**图14:**
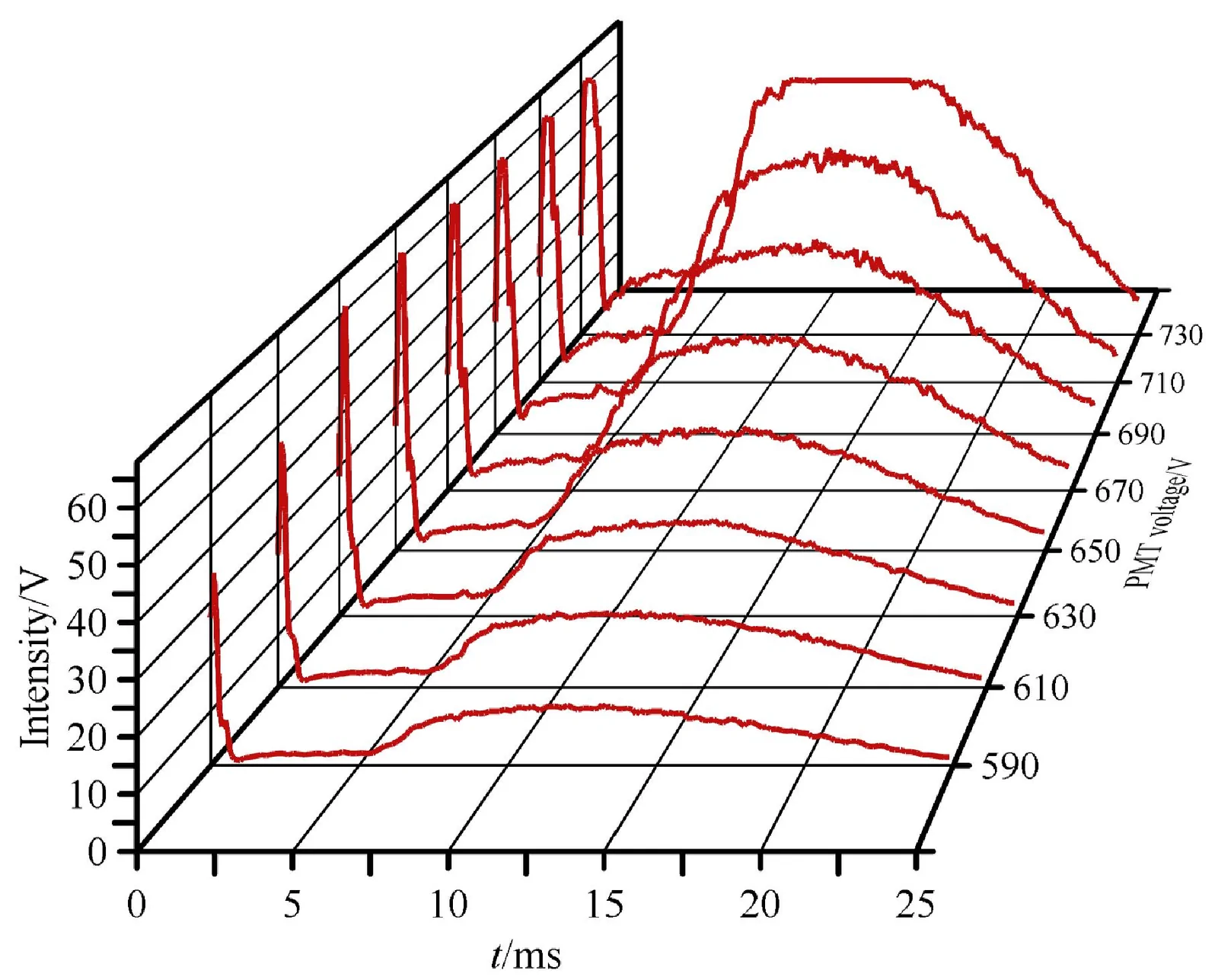
不同PMT电压下硫发光响应的三维叠加图

**图15:**
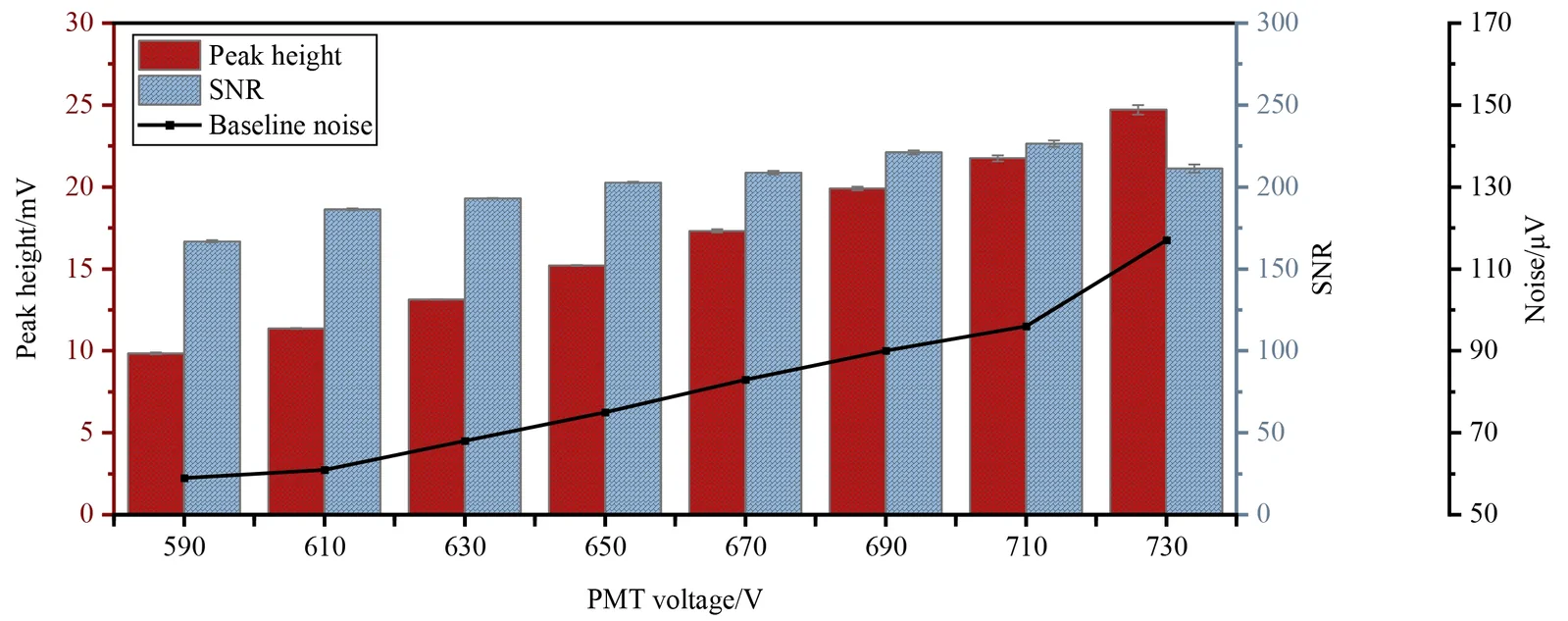
峰高、SNR和基线噪声随PMT电压的变化

此外，当PMT电压过低时，实验中观察到波形更新不连续现象。其原因在于低增益条件下，硫通道脉冲峰值不能稳定超过采集系统设定的触发电平，导致脉冲漏触发或数据更新不充分。

## 3 时间分辨选择性与硫延迟发光响应分析

在完成PFPD结构设计和运行参数优化的基础上，进一步分析其对硫信号与烃类背景实现时间分辨选择性的机理。PFPD 的选择性不仅与滤光片的波长选择有关，更重要的是源于不同发光物种在脉冲燃烧过程中时间分布特征的差异。本节结合H_2_S和C_4_H_10_的时间响应实验、火焰传播与自熄灭仿真结果，以及硫延迟发光反应过程，分析硫信号延迟窗口的形成原因。

### 3.1 烃类背景与硫信号的时间响应差异

为验证所研制PFPD对硫信号与烃类背景的时间分辨区分能力，在优选工作条件下分别测试了1.1 μmol/mol的H_2_S标准气体（底气为氮气）和10.3 μmol/mol的C_4_H_10_标准气体（底气为氮气）的延迟发光时间响应，结果见图16。

**图16:**
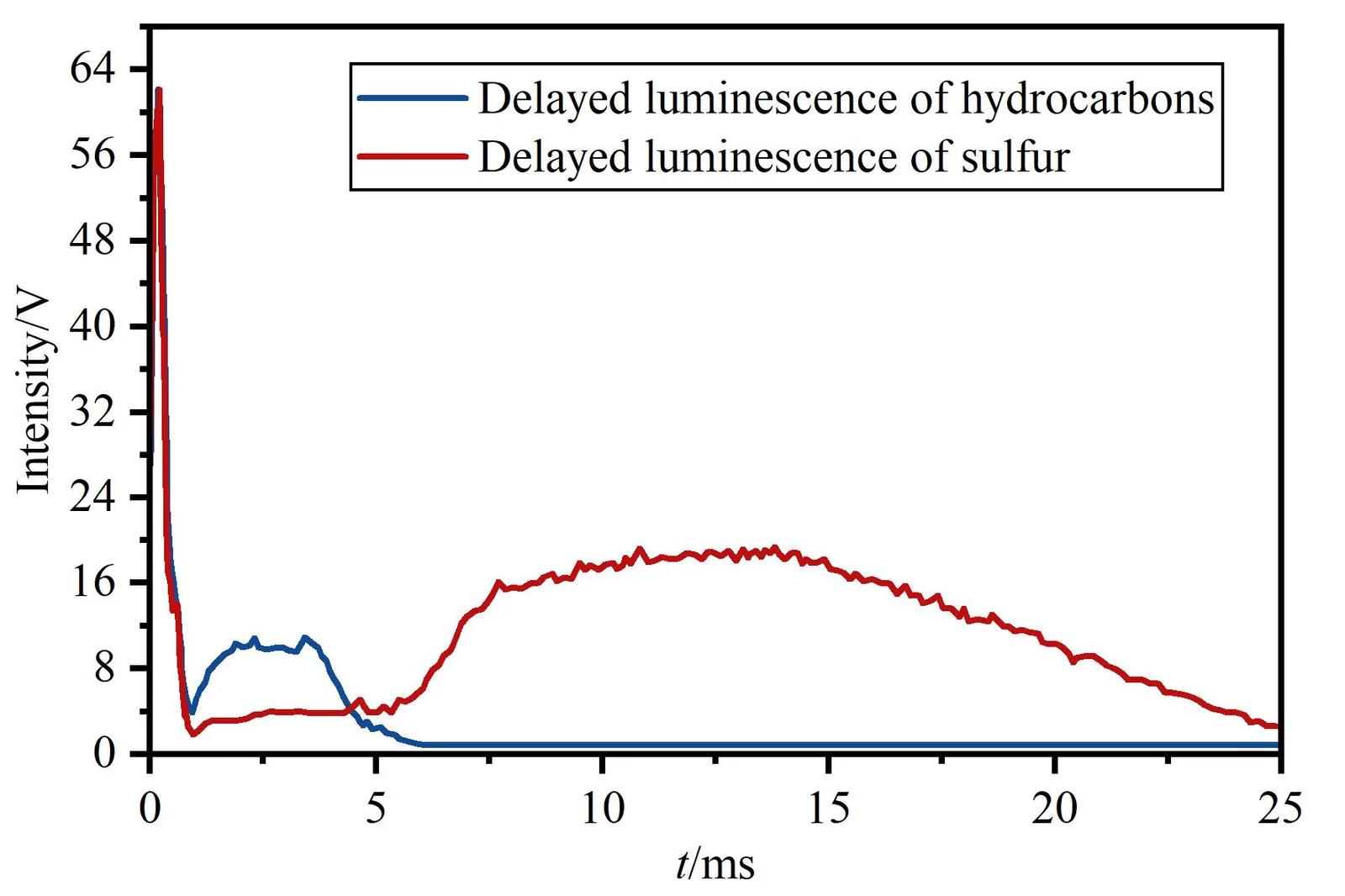
烃类与硫在PFPD中的延迟发光时间响应曲线

两种组分在0 ms附近均出现明显的瞬态峰，该信号主要来源于火焰在引火室点燃后，沿检测器上部空间向下传播，并在进入石英燃烧室上部区域时产生瞬时发光响应。除初始瞬态峰外，C_4_H_10_延迟发光主要分布在1~5 ms范围内，随后快速衰减；而H_2_S延迟发光主要分布在6~24 ms范围内，持续时间明显更长，表明硫与烃类背景在时间分布上具有显著差异。基于这一差异，通过设置合适的门延迟和积分窗口，可有效避开烃类背景的主要发光区间，同时保留硫延迟发光信号，说明该PFPD具备通过时间门控抑制烃背景干扰的能力。

### 3.2 主燃烧阶段与余焰区的时域对应关系

为进一步解释上述时间响应差异，将实验测得的发光时间分布与火焰传播和自熄灭仿真结果进行对应分析。前述仿真结果表明，脉冲火焰在燃烧室内完成轴向传播并发生自熄灭的时间约为1.97 ms；同时，监测点处·OH质量分数和温度在火焰前锋经过时迅速升高，并在数毫秒内快速衰减。该结果说明，PFPD中的主链式燃烧反应主要发生在点火后最初的几毫秒内，随后燃烧室进入无明显主火焰传播的高温余焰阶段。

实验响应与仿真结果具有较好的对应关系。烃类背景发光主要集中在1~5 ms区间，与主燃烧阶段在时间上基本重叠，说明烃类背景发光主要来自烃类分子在主燃烧阶段的裂解、氧化及短寿命发光物种的瞬态辐射。相比之下，H_2_S的特征发光主要出现在6~24 ms区间，明显滞后于主火焰传播与自熄灭过程，说明硫信号并不主要来源于火焰前锋本身，而是来源于主燃烧结束后余焰区内的后续含硫反应过程。

### 3.3 硫延迟发光窗口的形成机制

结合H_2_S和C_4_H_10_的时间响应差异以及火焰传播仿真结果可知，硫延迟发光窗口的形成与主燃烧阶段和余焰阶段中含硫物种的连续转化有关。在主燃烧早期，H_2_S随火焰前锋发生快速氧化，生成SO_2_等含硫中间产物；随着火焰自熄灭，烃类背景发光迅速衰减，而燃烧室内仍保持一定温度的余焰环境，使含硫中间体能够进一步转化并生成激发态S_2_^*^。该过程可用简化反应（1）~（4）表示：


$$H_{2}S+\frac{3}{2}O_{2}\to SO_{2}+H_{2}O$$ (1)

$$2SO_{2}+4H_{2}\to 4H_{2}O+S_{2}^{*}$$ (2)

$$S_{2}^{*}\to S_{2}+hv\;(\lambda \approx 394\;nm)$$ (3)

$$S_{2}^{*}+M\to S_{2}+M$$ (4)

其中，反应（1）表示在相对富氧的火焰区将H_2_S氧化为SO_2_；反应（2）表示SO_2_在富氢、轻度缺氧区域进一步转化并生成激发态S_2_^*^；反应（3）表示检测器实际采集到的硫特征发光；反应（4）表示激发态S_2_^*^与第三体M的碰撞失活的非辐射过程。

上述反应过程说明，硫延迟发光窗口的形成依赖于主燃烧结束后余焰区内含硫中间体的继续转化。因此，凡是影响余焰区温度状态、含硫中间体转化速率和S_2_^*^失活过程的因素，都会进一步改变延迟发光窗口的稳定性。其中，检测器温度是影响该过程的重要外部条件。

低温条件下，含硫中间体生成与后续转化受限，难以形成稳定的S_2_^*^发光区。当检测器温度约为150 ℃时，含硫前体生成、余焰区停留时间和S_2_^*^碰撞失活之间达到较优平衡，因此延迟发光平台和积分响应较好；继续升温后，局部当量比、余焰区停留时间及激发态物种失活过程可能发生变化，使有效进入S_2_^*^发光通道的比例下降，导致峰面积和灵敏度减小。此外，高温下1~3.5 ms区间的早期小峰有所增强，可能与火焰前缘短寿命发光物种及背景辐射有关，可通过合理设置门延迟和积分宽度加以抑制。

## 4 仪器性能测试

在完成引火条件、燃烧室供气参数、检测器温度及PMT电压等关键参数优化后，进一步在优选工作条件下验证PFPD的分析性能。本节主要对方法的线性范围、硫检出限和定量重复性进行评价，并与商业化硫检测器进行性能比对，以评价其痕量硫定量能力和运行稳定性。

### 4.1 测试条件与评价方法

为评价所研制PFPD的分析性能，实验采用GC-8860气相色谱仪（山东鲁南瑞虹化工仪器有限公司，中国）和H310（A）动态稀释仪（天津华翼科技有限公司，中国）。所用气体包括氮气、氢气和空气，纯度均不低于99.999%。测试用标准气体为10 μmol/mol和1.1 μmol/mol的H₂S，底气均为氮气。其中，采用动态稀释仪对10 μmol/mol的H₂S标准气体进行稀释，配制不同物质的量分数的测试气体，用于线性范围评价；1.1 μmol/mol的H₂S标准气体用于硫检出限和定量重复性测试。

色谱测试采用硫专用石英毛细管柱（30 m×0.32 mm×5.00 μm，山东鲁南瑞虹化工仪器有限公司，中国）。进样口温度为60 ℃，采用六通阀定量环无分流进样方式；载气为氮气，柱流速为1.5 mL/min；进样体积为0.125 7 mL。PFPD运行条件采用前述优化结果：引火室H_2_和空气流量分别为8.0 mL/min和19.0 mL/min，燃烧室H_2_和空气流量分别为5.0 mL/min和3.7 mL/min，检测器温度为150 ℃。时间门控参数为门延迟6 ms、门宽18 ms，触发电平为500 mV。

### 4.2 线性范围

采用不同含量的H_2_S标准气体，在相同进样条件下对硫通道的线性响应进行测定，结果见图17。结果表明，在所考察范围内，PFPD对H_2_S具有良好的线性响应，线性范围约覆盖2个数量级，与目前主流商业化色谱检测器相比，范围偏窄。

**图17:**
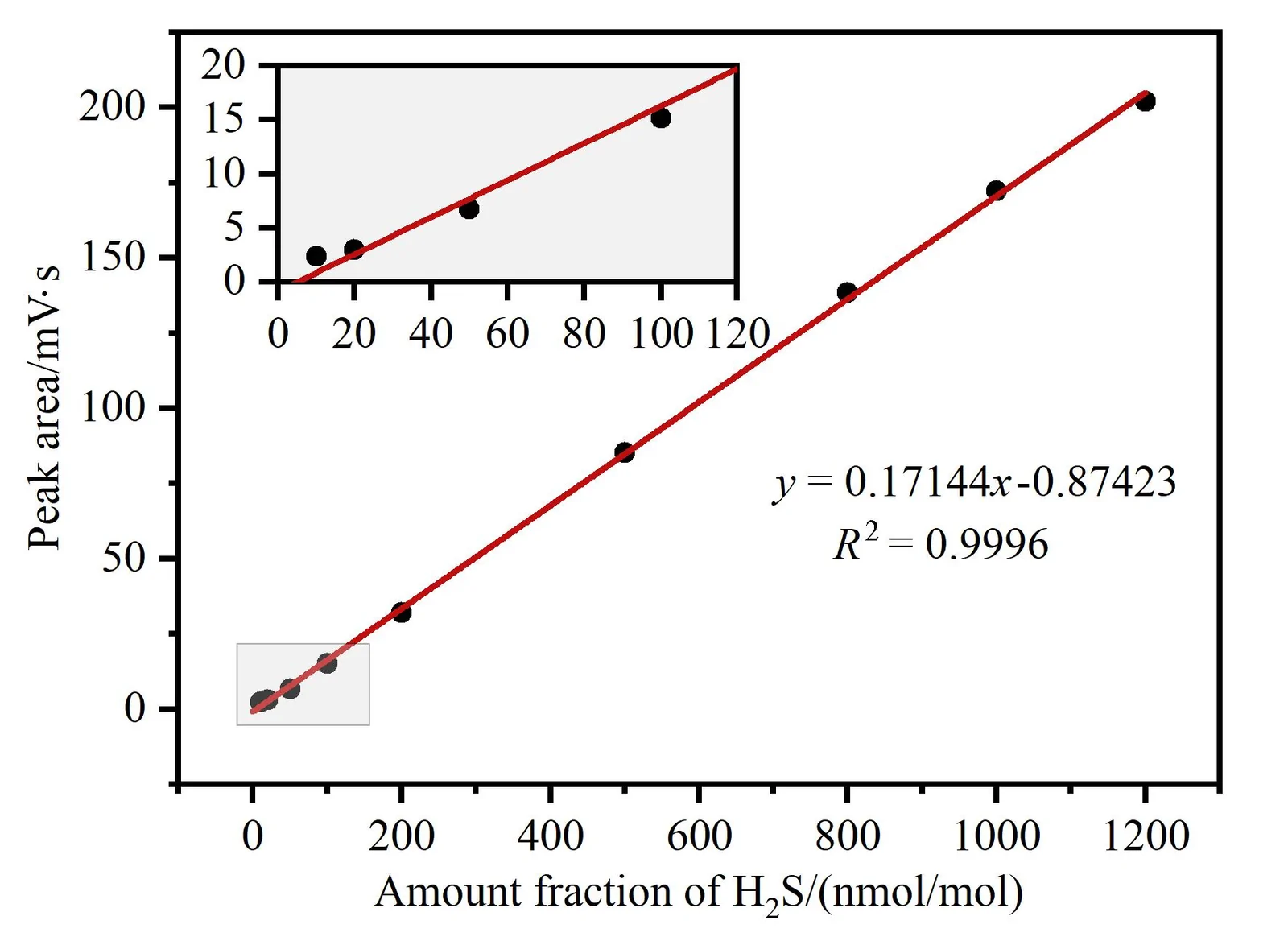
PFPD硫通道峰面积与H_2_S物质的量分数的线性拟合曲线

线性范围受限可能主要来源于两个方面。其一，信号探测链路的动态范围存在约束。在较高PMT增益条件下，实验中已观察到明显的信号“削顶”现象，说明输出信号开始偏离线性放大区，峰值信息受到压缩，从而限制了线性范围上限。其二，硫发光过程本身可能具有一定的非线性响应。在较高浓度条件下，激发态物种碰撞失活或自猝灭作用还可能出现发光饱和，导致响应增幅减弱。后续研究将优先从多量程采集、信号衰减控制、双门采样及门控响应比校正等方面入手，拓展较大进样量条件下的线性响应范围，并进一步评估激发态S_2_^*^的自猝灭效应是否成为较大进样量的限制因素。

### 4.3 硫检出限

以H_2_S标准气体为测试对象，经动态稀释后采用六通阀定量环进样，记录硫的峰高及峰宽等参数，并结合基线噪声计算检出限。本研究采用行业通用的PFPD检出限计算公式（式（5）），该公式来源于文献［^18^］，为脉冲火焰光度检测器硫检出限测试的标准方法。

实验中采用1.1 μmol/mol H_2_S标准气体，经动态稀释仪稀释30倍后进行测试。进样体积为0.1257 mL，连续进样7次，由工作站提取各次进样对应的峰高和峰宽参数，并取其代表值用于检出限计算：


$$D_{PFPD}=\sqrt[{}]{\frac{2N(Wn_{S}{)}^{2}}{h(W_{1/4}{)}^{2}}}$$ (5)

式中，*D*_PFPD_：PFPD对硫的检出限（g/s）；*N*：基线噪声（μV），取*N*=21 μV；*W*：硫化氢的进样量（g），取*W*=6.42×10^-12 ^g；*h*：硫的峰高（μV），取*h*=94.57 μV；*W*_1/4_：硫的峰高1/4处的峰宽，取*W*_1/4_=9.92 s； *n*_S_：硫化氢分子中硫原子占的比例，*n*_S_=0.9407。

由式（5）计算得到，*D*_PFPD_=4.1×10^-13^ g/s。

### 4.4 定量重复性

将1.1 μmol/mol H_2_S标准气体连续7次进样，结果见图18。各次进样峰形和保留时间保持稳定，峰面积RSD为0.37%，说明该检测器在推荐工作条件下具有良好的定量重复性和运行稳定性。

**图18:**
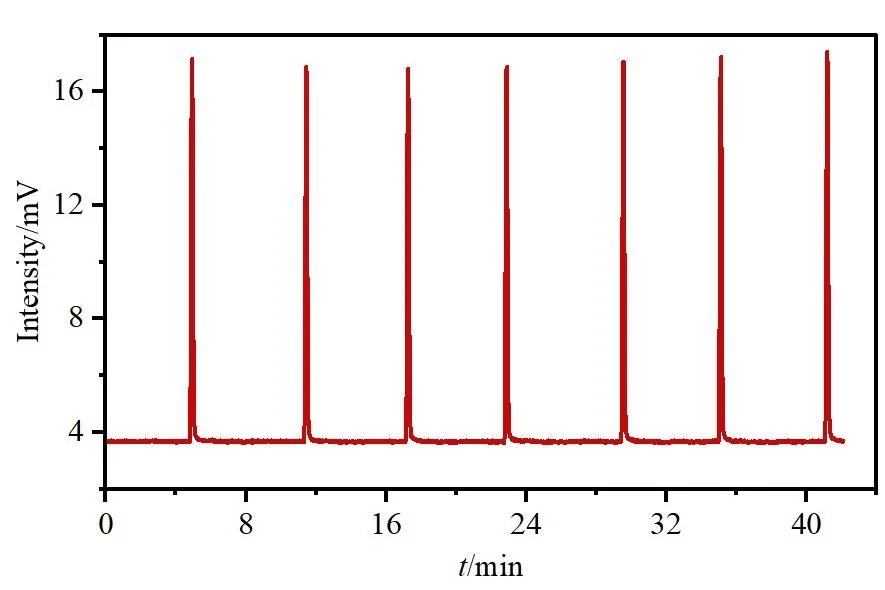
1.1 μmol/mol H_2_S连续7次进样的色谱图

综合以上结果可知，该PFPD在优化工作条件下具备较高灵敏度、良好线性响应和较高重复性，表明其已具备稳定的痕量硫定量检测能力，并为后续复杂基体应用奠定了性能基础。

### 4.5 与商业化仪器的性能对比

为进一步评价自研PFPD的检测性能，将本文所得结果与典型商业化硫选择性检测器的公开性能指标进行对比，结果见表4。

**表4:** 本研究PFPD与商业化硫检测器主要性能指标对比

Detector	Sulfur detection limit/（g/s）	Linear range	Quantitative repeatability
PFPD developed in this work	4.1×10^-13^	2 orders of magnitude	0.37% （1.1 μmol/mol H₂S）
5383 PFPD^［17，19］^	1×10^-12^	2.4 orders of magnitude	11% （0.657 μmol/mol H₂S）
Nexis FPD-2030^［20，21］^	2×10^-12^	3 orders of magnitude	0.91% （1 μmol/mol thiophene）

在优化工作条件下，自研PFPD的硫检出限为4.1×10^-13^ g/s，低于表中两款商业化仪器的公开标称检出限，说明该检测器具有较好的痕量硫检测能力。定量重复性实验结果表明，自研PFPD对H_2_S的峰面积重复性低至0.37%，能够满足痕量硫化物定量分析对信号稳定性的基本要求。上述结果表明，通过燃烧气路解耦、检测器温度优化及时间分辨采集窗口设置，可以有效提高硫延迟发光信号的采集稳定性和重复性。

但同时也应看到，自研PFPD的线性范围为2个数量级，略窄于两款商业化仪器。结合前文分析，该限制一方面来源于硫发光过程本身可能具有一定的非线性响应，另一方面受限于当前单量程信号采集链路的动态范围，在较大进样量下，PMT及采集链路易出现信号饱和（“削顶”），进而限制线性范围上限，这也是后续检测器优化的核心方向。

总体而言，在本研究测试条件下，自研PFPD表现出较低的硫检出限和良好的定量重复性。与表4所列商业化硫选择性检测器的公开指标相比，部分性能指标达到相当或较优水平。鉴于各项数据的测试对象、物质的量分数及实验条件存在差异，该比较主要用于性能量级参考，不构成严格的横向性能评价。自研PFPD仍具有进一步工程优化的潜力，后续可通过多量程信号采集和双门控检测等方法拓宽线性范围，进一步提升仪器的综合性能。

## 5 结论

本文针对PFPD在痕量含硫化合物检测中存在的参数耦合强、复现性依赖经验调参等问题，提出了以“双路EPC解耦供气”为核心的改进方案，并完成了PFPD结构设计、运行参数优化、硫延迟发光响应分析及仪器性能测试。结果表明，通过将引火室点火基准与燃烧室分析工况分离控制，可有效降低气路参数联动带来的不确定性，使点火稳定性、余焰区调控和增益匹配之间的关系更加清晰，从而提高系统运行的可控性与可解释性。

在优化条件下，所研制的PFPD获得了较好的硫延迟发光响应、重复性和检测性能。与传统依赖主燃烧气与旁路气协同调节的供气方案相比，本研究通过解耦控制将难以独立优化的问题拆解为可分步调控的过程，为识别影响硫选择性响应的关键限制因素提供了更清晰的路径。该结果表明，“解耦调控-参数窗口化”的思路不仅适用于本仪器，也为不同仪器结构和应用条件下较优硫响应窗口的复现提供了参考。

从应用角度看，所研制的PFPD具有用于复杂烃类基体中痕量硫化物检测的潜力，可为天然气、炼化过程气、工业尾气及恶臭排放等场景中的硫选择性检测提供参考。特别是在需要兼顾高灵敏度、较好重复性和仪器结构自主可控的应用中，双路供气解耦和窗口化参数优化方法具有进一步工程化发展的价值。

同时，本研究仍存在一定局限。当前性能验证主要基于H_2_S标准气体，复杂基体和多组分硫化物体系中的选择性、抗干扰能力和长期稳定性仍需进一步考察；硫延迟发光响应分析主要基于宏观时间响应和仿真结果，对燃烧区中关键自由基、激发态S_2_^*^及猝灭过程的直接诊断仍不充分；此外，线性动态范围仍有进一步拓展空间。后续研究可结合火焰可视化诊断、反应动力学模拟、复杂样品测试、多量程信号采集和自适应门控校正，进一步完善PFPD燃烧调控机理并提升其工程化应用能力。
